# From Modular to Centralized Organization of Synchronization in Functional Areas of the Cat Cerebral Cortex

**DOI:** 10.1371/journal.pone.0012313

**Published:** 2010-08-26

**Authors:** Jesús Gómez-Gardeñes, Gorka Zamora-López, Yamir Moreno, Alex Arenas

**Affiliations:** 1 Instituto de Biocomputación y Física de Sistemas Complejos (BIFI), Universidad de Zaragoza, Zaragoza, Spain; 2 Departamento de Matemática Aplicada, Universidad Rey Juan Carlos (ESCET), Móstoles (Madrid), Spain; 3 Interdisciplinary Center for Dynamics of Complex Systems, University of Potsdam, Potsdam, Germany; 4 Departamento de Física Teórica, Universidad de Zaragoza, Zaragoza, Spain; 5 Departament d'Enginyeria Informática y Matematiques, Universitat Rovira i Virgili, Tarragona, Spain; Indiana University, United States of America

## Abstract

Recent studies have pointed out the importance of transient synchronization between widely distributed neural assemblies to understand conscious perception. These neural assemblies form intricate networks of neurons and synapses whose detailed map for mammals is still unknown and far from our experimental capabilities. Only in a few cases, for example the *C. elegans*, we know the complete mapping of the neuronal tissue or its mesoscopic level of description provided by cortical areas. Here we study the process of transient and global synchronization using a simple model of phase-coupled oscillators assigned to cortical areas in the cerebral cat cortex. Our results highlight the impact of the topological connectivity in the developing of synchronization, revealing a transition in the synchronization organization that goes from a modular decentralized coherence to a centralized synchronized regime controlled by a few cortical areas forming a Rich-Club connectivity pattern.

## Introduction

Processing of information within the nervous system follows different strategies and time-scales. Particular attributes of the sensory stimuli are transduced into electrical signals of different characteristics, e.g. regular or irregular spiking, the rate of firing, etc. Further aspects of the information are “encoded” by specialization of neurons, e.g. the color and orientation of a visual stimulus will activate only a set of neurons and leave others silent. For higher order processes such as feature binding and association, the synchronization between neural assemblies plays a crucial role [1]–[5]. For example, subliminal stimulation which is not consciously perceived, triggers a similar cascade of activation in the sensory system but fails to elicit a transient synchronization between distant cortical regions [6].

The neurons comprising the nervous system form a complex network of communications. To what extent this intricate architecture supports the richness and complexity of the ongoing dynamical activity in the brain is a fundamental question [7]. A detailed map of the neurons and their synapses in mammals is still unknown and far from our experimental capabilities. Only in a few cases, for example the nematode *C. elegans*, we know the complete mapping of the neuronal tissue. In the cases of macaque monkeys and cats a mesoscopic level of description is known, composed of cortical areas and the axonal projections between them. These areas are arranged into modules which closely follow functional subdivisions by modality [8]–[13]. Two cortical areas are more likely connected if both are involved in the processing of the same modal information (visual, auditory, etc.) Beyond this modular organization, some cortical areas are extensively connected (referred as *hubs*) with projections to areas in all modalities [14], [15]. For the corticocortical network of cats, these hubs are found to be densely interconnected forming a *hidden* module [16], at the top of the cortical hierarchy which might be responsible for the integration of multisensory information. A core of cortical areas has also been detected in estimates of human corticocortical connectivity by Diffusion Spectrum Imaging [17]. Following the above discussion that synchronization plays a major role in the processing of high level information, it would be important to analyze the synchronization behavior of these networks in relation to their modular and hierarchical organization. To this end, we simulate the corticocortical network of the cat by non-identical phase oscillators and we follow the evolution of their synchronization from local to global.

The study of synchronization phenomena is a useful tool to analyze the substrate of complex networks. The dynamical patterns under different parameters unveil features of the underlying microscopic and mesoscopic organization [18]. In particular, recent studies highlight the impact that the topological properties such as the degree heterogeneity, the small-world effect and the modular structure have on the path followed from local to global synchronization [18]–[21].

**Figure 1:**
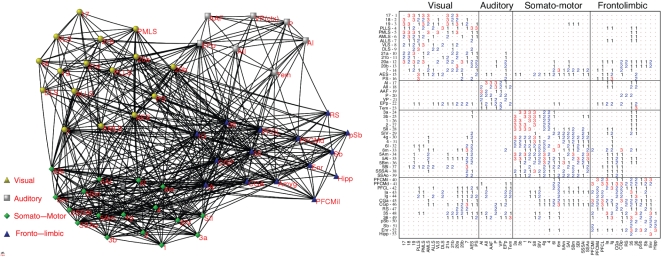
The brain cortical network of the cat. On the left we show the topology of the nodes (areas) and links (axon interconnections) between them. On the right the weighted adjacency matrix is shown. The weight of the links denote the axon density between two connected areas. Besides the matrix shows the partition of the network into four main modules of modally-related areas: Visual, Auditory, Somatosensory-Motor and Fronto-Limbic.

In this work we study the routes to synchronization in the corticocortical network of cats brain (see Figure 1) by modelling each cortical area as a phase oscillator with an independent internal frequency. This assumption considers that the ensemble of neurons contained within a cortical area behaves coherently having a well defined phase average whose dynamics is described by the internal frequency [22]. The coupling between areas is modelled using the Kuramoto nonlinear coupling and its relative strength can be conveniently tuned to allow for the observation of synchronization at different scales of organization. This seemingly crude approximation allows to obtain similar synchronization patterns as those observed with more refined models based on neuronal ensembles placed at each cortical area [23]–[25]. For instance, using the Kuramoto model one obtains that highly connected areas promote synchronization of neural activity just as revealed by the more stylized model used for the dentate gyrus [26].

Our results point out that complex structures of highly connected areas play a key role in the synchronization transition. In contrast to the usual partition of the brain cortex into four sets of modally-related areas, we find that this modular organization plays a secondary role in the emergence of synchronization patterns. On the contrary, we unveil that a new module made up of highly connected areas (not necessarily modally-related) drives the dynamical organization of the system. This new set is seen as the Rich-Club of the corticocortical network. Surprisingly, the new partition of the network including the Rich-Club as a module preserves the modular behavior of the system's dynamics at low intercortical coupling strengths. This modular behavior transforms into a centralized one (driven by the Rich-Club) at the onset of global synchronization highlighting the plasticity of the network to perform specialized (modular) or integration (global) tasks depending on the coupling scale.

## Results

As introduced above, we describe the dynamical behavior of the cortical network using the Kuramoto model [27], where the time evolution of the phase of each cortical area, 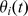, is given by
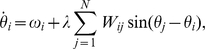
(1)where 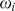 is the internal frequency associated to area 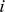 and 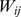 is the weighted inter-cortical coupling matrix that takes a value 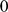 if area 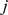 does not interact with the dynamics of area 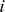 while 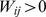 otherwise. In this latter case 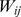 can take values 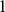, 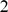 or 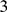 depending on the axon density going from area 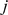 to area 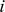. Let us remark that the inter-cortical coupling matrix is not symmetric so that in general 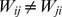. Besides, the inter-cortical dynamical coupling is modeled as the sine of the phase differences between two connected areas such that when 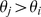 the average phase of area 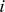 accelerates while that of area 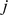 slows down to approach each other. Finally, the parameter 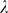 accounts for the strength of the inter-cortical coupling.

In a system composed of all-to-all coupled oscillators, the Kuramoto model shows a transition from incoherent dynamics to a synchronized regime as 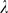 increases [28], [29]. However, when the system has a nontrivial underlying structure this transition does not take place in an homogeneous manner. In complex topologies, and for moderate coupling values, certain parts of the system become synchronized rather fast whereas other regions still behave incoherently. Therefore, one can monitor the synchronization patterns that appear as the coupling 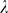 is increased and describe the path to synchronization accurately [18] by reconstructing the synchronized subgraph composed of those nodes and links that share the largest degree of synchronization (see Materials and Methods). The study of these synchronization clusters as the coupling 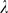 is increased allow to unveil the important set of nodes that drives these dynamical processes in the system.

We will analyze different scales of organization: 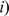 the macroscopic scale referring to global synchronization of the network; 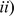 the microscopic scale of organization corresponding to the individual state of the oscillators; and finally 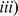 the intermediate mesoscopic scale of dynamical organization between the macroscopic and microscopic scales. Usually, it consists of groups of nodes classified according to a certain additional information, for example that provided by the anatomico-functional modules. Every scale of organization requires a particular set of statistical descriptors. This is especially important in the mesoscopic scale where changing the groups, the characterization of the system is also changed.

### Macroscopic analysis

We start by describing how global synchronization is attained as the inter-cortical coupling 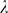 is increased. Global synchronization is characterized by the usual Kuramoto order parameter, 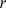, and the fraction of links that are synchronized 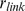 [20], [21] (see Materials and Methods). Both parameters take values in the region 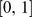, being close to 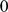 when no dynamical coherence is observed and close to 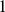 when the system approaches to full synchronization.

**Figure 2:**
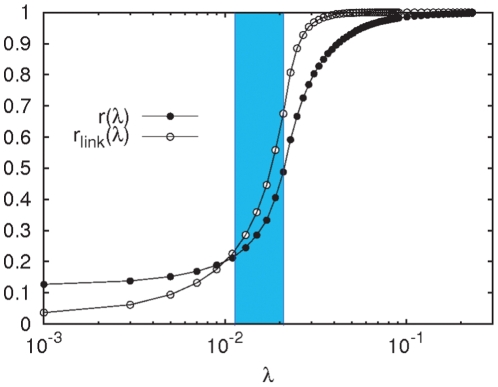
Synchronization diagrams. The figure shows the evolution of the Kuramoto order parameter 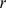 and the fraction of synchronized links 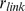 as the coupling strenght is increased. The transition from asynchronous dynamics to global dynamical coherence as 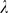 grows is clear from the two curves. The region in blue corresponds to the onset of synchronization.

In Fig. 2 we show the evolution of 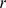 and 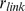 as a function of 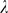. The plot reveals a well defined transition from incoherent to globally synchronized, the onset of synchronization occurring for coupling strength 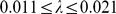. When 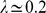, the system reaches the fully synchronized state. In the following, we will explore this transition in more detail and at lower scales of dynamical organization.

### Mesoscopic analysis as described by the four anatomical modules

The measures 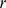 and 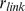 describe completely the dynamical state of the system if one assumes that all the cortical areas behave identically. However, it is possible to extract more information about the local dynamical properties of the system. In particular, for a given value of 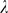 we can monitor the degree of synchronization between two given areas 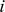 and 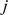, 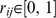 (see Materials and Methods).

The studies of the transition to synchronization in modular architectures [19], [21] show that synchrony patterns appear first at internal modules, *i.e.* synchrony shows up among the nodes that belong to the same module due to a larger local density within the module and similar pattern of inputs of the nodes. As the coupling 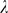 is increased, synchrony starts to affect the links connecting nodes of different clusters and finally spreads to the entire system. Now, we analyze whether the four anatomico-functional modules of the corticocortical network of the cat act also as dynamical clusters in the synchronization transition. To this end, we have analyzed the evolution of the average synchronization within and between the four anatomical modules taking into account solely the information about the dynamical coherence 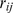 between the network's areas. We define the average synchronization between module 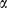 and module 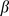 as:
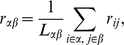
(2)where 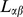 is the number of possible pairs of areas from modules 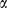 and 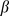, *i.e.*, 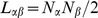 where 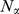 and 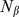 are the number of areas of modules 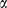 and 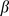 respectively. If 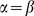 equation (2) denotes the intramodule average synchronization.

**Figure 3:**
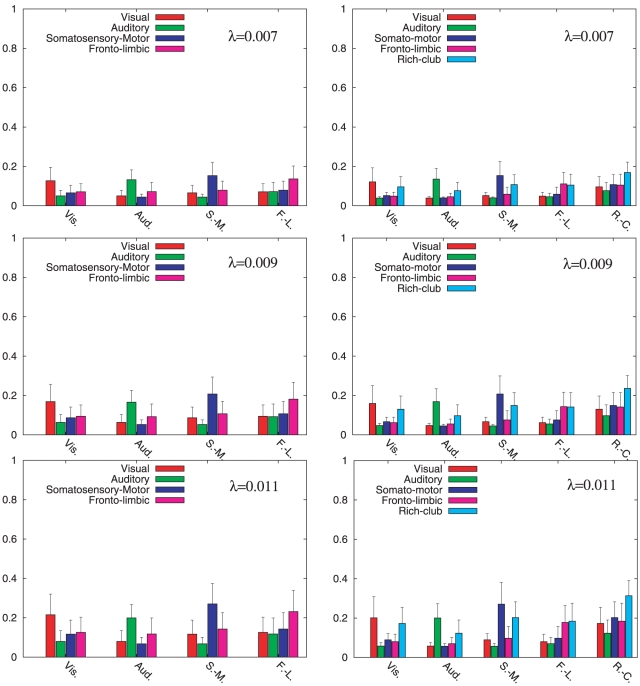
Dynamical correlation within the 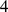 modal clusters (left column) and the new 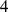 modally-related clusters and the Rich-Club (right column) before the onset of synchronization. The bars of the histograms show the values of the dynamical correlation 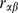 (see Equation (2)) between the 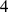 original modules (left) and the new 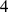 clusters and the Rich-Club (right). From top to bottom we show the cases for 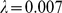, 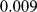 and 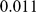 that correspond to the region before the onset of synchronization.

**Figure 4:**
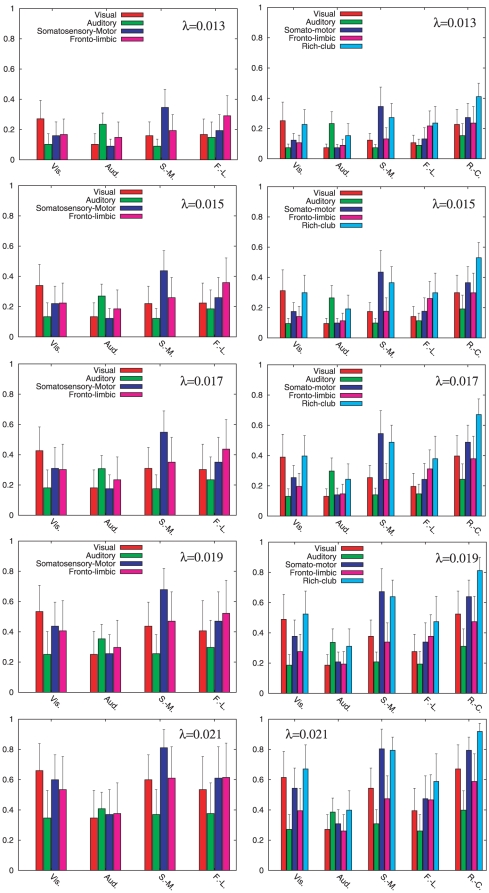
Dynamical correlation within the 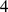 modal clusters (left column) and the 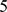 dynamical clusters (right column) at the onset of synchronization. The bars of the histograms show the values of the dynamical correlation 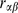 (see Equation (2)) between the 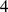 modules (left) and the new 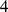 modally-related clusters and the Rich-Club (right). From top to bottom we show the cases for 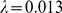, 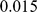, 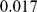, 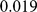 and 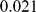 that correspond to the onset of synchronization.

The histograms in the left columns of Figure 3 and Figure 4 show the values of the set 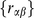 for several values of 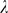 corresponding to the region before (Fig. 3) and at (Fig. 4) the onset of synchronization. From the histograms it is clear that the degree of synchronization grows with 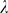 as it occurs for the global parameters 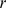 and 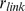 in the macroscopic description. Besides, the histograms inform us about the importance of the anatomical partition in the dynamical organization of the cat cortex. From Figure 3 it becomes clear that the average dynamical correlation within areas of the same anatomical module is higher than that between areas belonging to different modules. Moreover, before the onset of synchronization, for 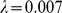 to 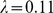 all the modules satisfy 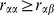 for 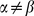. At the onset of synchronization (left column of Figure 4) we observe that the initial intra-module synchronization is progressively compensated by the increase of inter-module dynamical coherence. In particular, the influence of the Somatosensory-Motor on the remaining modules is remarkably relevant during the onset of synchronization and, for 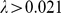, this module shows the largest degree of synchronization with the rest of the system.

### Microscopic analysis: Unveiling the dynamical organization

The mesoscopic analysis based on the partition of the cortex into four modules has revealed a fingerprint of a hierarchical organization of the synchronization based on the dominating role of the Somatosensory-Motor module. Here we will analyze the microscopic correlation between all the areas of the cortical network to unveil whether there is a group of nodes that lead the onset of synchronization in the system. To this purpose we study the subgraphs formed by those pairs of areas sharing an average synchronization value 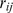 larger than a threshold 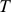. Certainly when 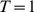 the subgraph is the null (empty) graph and for 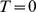 the subgraph is the whole cortex.

**Figure 5:**
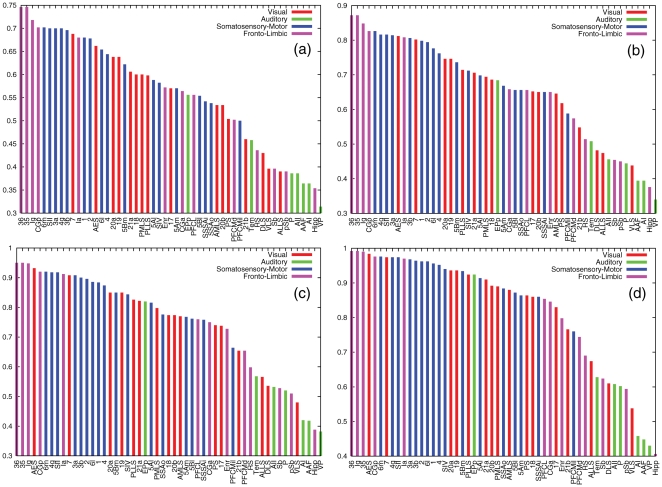
Synchrony Rank of areas: Unveiling anatomical structure of the largest synchronized areas. In these plots we show the rank of areas from the most to the less synchronized for 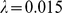 (a), 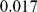 (b), 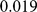 (c) and 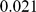 (d). The height of the bars account of the maximum value of the threshold, 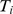, at which the area is incorporated in the synchronized subgraph. Besides, the colour of each bar accounts for the corresponding module of the cortical area.

In Figure 5 we show a ranking of the cortical areas at coupling strengths 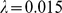, 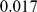, 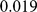 and 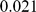 corresponding to the onset of synchronization. The rankings are made by labeling the area 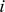 with the largest value of the threshold at which the area is incorporated into the synchronized subgraph as 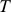 is tuned from 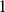 to 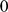. Additionally, the modular origin of the areas has been color coded to distinguish the role of each module. From the rankings we find that there are three areas *36*, *35* and *Ig*, from the Fronto-Limbic system, that share the largest degree of synchrony. In all the cases, several jumps in the threshold are observed that distinguishes those groups of cortical areas that are more synchronized than the rest of the network. For instance, at 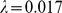 we observe 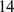 areas spanning from the *36* area to the *2* (Somatosensory-Motor system) while for 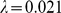 we find 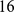 areas ranging from the *36* to the area *4* (Somatosensory-Motor system). From these figures it is no clear that there is one module dominating the synchronization. Quite on the contrary both Somatosensory-Motor and Fronto-Limbic systems are well represented among the most synchronized areas.

**Figure 6:**
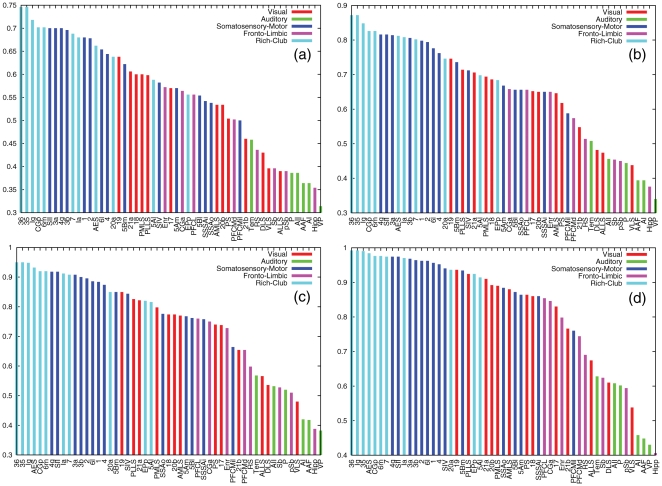
Synchrony Rank of areas: Structure of the largest synchronized areas as described by the Rich-Club. The two plots show the same synchrony ranks as in Figure 5 (again 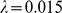 (a), 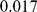 (b), 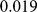 (c) and 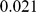 (d)). We have recolored the bars of those areas corresponding to the Rich-Club to highlight the dominant role of this topological structure in the composition of the largest synchronized areas.

A further analysis of the composition of these highly synchronized areas reveals that most of them take part in a higher-order topological structure of the cortical network: a Rich-Club (see Materials and Methods). The Rich-Club of a given network is made up of a set of nodes with high connectivity, which at the same time, form a tightly interconnected community [30], [31]. Therefore, the Rich-Club of a network can be described as a highly cohesive set of hubs, that form a dominant community in the hierarchical organization. The Rich-Club of the cat cortex is composed of 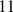 cortical areas of different modalities: 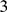 visual areas (*20a*, *7* and *AES*), 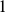 area from the Auditory system (*EPp*), 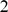 areas of the Somatosensory-Motor system (*6m* and *5Al*) and 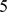 fronto-limbic areas (*Ia*, *Ig*, *CGp*, *35* and *36*). In Figure 6 we show again the ranking of areas for the cases 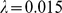, 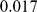, 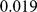 and 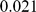 but highlighting those areas belonging to the Rich-Club in black. From the plots it is clear that most of the Rich-Club areas are largely synchronized. In particular for 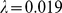 and 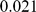 the 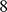 out of the 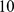 largest synchronized areas of the network belong to the Rich-Club, although originally they belong to the Fronto-Limbic, Somatosensory-Motor and the Visual systems in the partition into four modules.

### Mesoscopic analysis of synchronization including the Rich-Club

Looking at the composition of the Rich-Club we observe that it is mainly composed of fronto-limbic areas. Taking into account that we previously observed how the Somatosensory-Motor system took the leading role within the description with 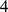 modules of the synchronization transition, this dominance of the Fronto-Limbic system in the Rich-Club may seem counterintuitive. To test the role of the Rich-Club in the synchronization transition we define a new partition of the cortical network into 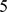 clusters composed of the Rich-Club (as defined above) and the 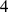 original modules, but with the corresponding areas of the Rich-Club removed from them.

At the mesoscopic scale, we investigate the self-correlation of the new five clusters and their cross correlation according to Equation (2). In the right columns of Figure 3 and Figure 4 we present the histograms of the inter and intra correlations for different values of the coupling. In both figures the role of the Rich-Club orchestrating the process towards synchrony while increasing the coupling strength becomes clear. More importantly, the addition of the Rich-Club to the partition helps to elucidate the patterns of synchrony: both the dynamical self-correlation of the four modally-related clusters and their correlation with the Rich-Club remain large. In particular, we observe in Figure 3 that, before the onset of synchronization, these new five modules keep a large self-correlation during this stage. On the other hand, at the onset of synchronization (Figure 4) the four modally-related clusters loose their large self-correlation in the following sequence: The “Fronto-Limbic” cluster remains autocorrelated until 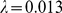, the “Visual” one until 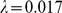, the “Auditory” system until 0.019 and the “Somatosensory-Motor” cluster until 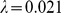. For larger couplings, all the clusters switch from autocorrelation to be synchronized with the Rich-Club, which acts as a physical mean-field of the system. Moreover, during the whole synchronization path the Rich-Club is always the cluster with the largest self-correlation. Thus, the distinction of Rich-Club in the partition preserves the autocorrelation of the four modal clusters before the onset of synchronization while, at the same time, rules the path to complete dynamical coherence during the onset of synchronization. This two-stage dynamics (modal cluster synchronization followed by a sequential synchronization with the Rich-Club) supports the idea that the modular organization with a centralized hierarchy described in [16] facilitates the segregation and integration of information in the cortex.

### Characterization of the transition from modular to centralized synchronization

The results so far indicate a plausible transition from modular to centralized organization in the cortex, depending on the coupling strength. In particular, we have shown patterns of synchronization that change the behavior while increasing the coupling 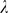. Now we propose a characterization of this change in terms of statistical descriptors. To this end, we define two different measures: *(i)* the dynamical modularity (DM) and *(ii)* the dynamical centralization (DC). The dynamical modularity compares the degree of internal synchrony within the clusters with the average dynamical correlation across clusters. With this aim we define the DM as the fraction of the average self-correlation of clusters and the average intercluster cross-correlation. For a network composed of 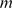 clusters we have:
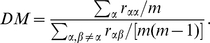
(3)The DM will take values above 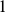 when the system contains true dynamical clusters while 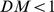 means that the entitity of the partition is not consistent with a clustered behavior. On the other hand, the dynamical centralization of the network measures the relative difference in synchrony between the maximum among the 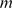 clusters of 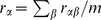 and the average degree of synchrony over clusters, 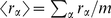:
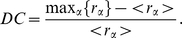
(4)In the case of the DC we always obtain positive values. A large value of DC means that the system displays a highly centralized dynamical behavior around a leading cluster while we will obtain 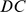 values approaching to 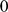 when the system behaves homogeneously, *i.e.* when there is no leading cluster that centralizes the dynamics.

**Figure 7:**
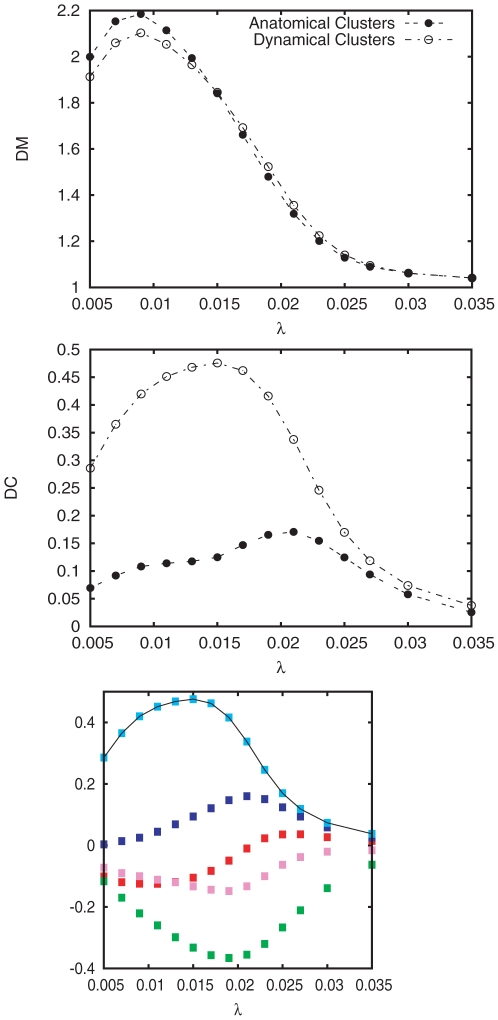
Transition from Modular to Centralized organization of synchronization. The two upper plots show the evolution of the dynamical modularity 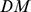 and the dynamical centralization 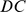 as a function of 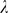. Both panels show the evolution of the above properties for the network described by means of both the original 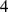 modal clusters and the 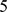 new modules including the Rich-Club. The panel in the bottom shows the evolution of the DC of the 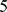 new modules obtained by replacing in Equation (4) the term 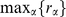 by each 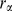 corresponding to the new 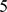 modules. From the curves it is clear that the dynamics is centralized around the Rich-Club.

We have measured both DM and DC for the original partition into 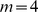 modules and the new partition with 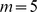 incorporating the Rich-Club. In Figure 7 we show the evolution of the two quantities as a function of the coupling parameter. For the case of the DM we confirm that the partition with the Rich-Club keeps the modular behavior of the original partition along the whole synchronization path. The DM is remarkably high for low values of the coupling 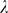 pointing out that before the synchronization onset the internal synchronization dominates over the cross-correlation between the clusters. Regarding the DC we find that in both partitions 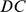 increases with 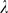 reaching a maximum around the synchronization onset, signaling that at this point the synchronization is driven hierarchically and lead by one of the modules. However, the partition that incorporates the Rich-Club shows the remarkably larger values of DC along the whole path, specially around the synchronization onset. In particular, the dominant role of the Rich-Club is clearly highlighted by the maximum of the DC at 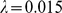, just at the onset of synchronization. In order to verify that the Rich-Club is the cluster contributing to the term 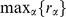 in the dynamical centrality of the network we plot in Figure 7 the evolution of the DC considering each of the 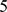 modules as the the central cluster by substituting 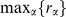 by the corresponding value of 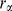. From the plot it is clear that the Rich-Club is the central cluster orchestrating the dynamics of the system at the onset of synchronization.

The coupled evolution of DM and DC corroborates the two-mode operation of the cortical network when described with the Rich-Club and the remaining parts of the four original modules: At low values of the coupling, the modular structure of the network dominates the synchronization dynamics, pointing out the capacity to concentrate sensory stimuli within its corresponding module. When the coupling is increased the dynamical organization is driven by a leading subset of nodes, organized in a topological Rich-Club, that integrates information between different regions of the cortex.

## Discussion

Previous simulations performed in the cat cortical network [23]–[25] have dealt with its synchronization properties. In these works, the transition towards synchronization is studied by using ensembles of neurons coupled through a small-world topology placed inside each cortical area whereas different neuronal populations are dynamically coupled accordingly to the topology of the cat cortical network. By means of this two-level dynamical model, numerical simulations allowed to find different clusters of synchrony as the coupling between the cortical areas is increased. It was found that only for weak coupling these clusters were closely related to the four modal clusters. In the light of these previous studies, and the recent report of a novel modular and hierarchical organization of the corticocortical connectivity [16], the issue regarding the relation between the mesoscopic structure of the cat cortex and its dynamical organization remains open.

Here, we have investigated the evolution of synchronization in a network representing the actual connectivity among cortical areas in the cat's brain. We have confirmed, that the role of the different areas in the path towards synchrony is difficult to assess using the traditional partition into four groups of modally-related areas. On the contrary, we have shown that a subset of areas, forming a topological Rich-Club, orchestrates this process. The distinction of this subset permits the interpretation of a new mesoscale formed by the four modules, excluding some nodes that form the Rich-Club, which are considered here as the fifth module. This proposed structure allows us to reveal a transition in the path to synchronization as a function of the coupling strength, that seems to indicate a two-mode operation strategy. For low values of the coupling, a state of weak internal coherence within the five modules governs the coordination dynamics of the network. As the coupling strength is increased, the Rich-Club becomes the responsible of centralizing the network dynamics and leads the transition towards global synchronization.

Finally, the composition of the Rich-Club allows to make some additional biologically relevant observations. First, the Rich-Club comprises of cortical areas of the four different modalities, supporting the hypothesis of distributed coordination dynamics at the highest levels of cortical processing such as integration of multisensory information [32]. Second, the Rich-Club comprises of most of the frontal areas in the Fronto-Limbic module. Moreover, the areas of the Rich-Club collected from the original Somatosensory-Motor system contain the so-called supplementary motor area (SMA). The SMA is a controversial region of the motor cortex, since in contrast with the rest of somatosensory-motor areas it is in charge of the initiation of planned or programmed movements [33]. Furthermore, the area *AES* of the Rich-Club, originally assigned to the Visual module, is believed to integrate all visual and even auditory signals for their multimodal processing and transference as coherent communication signals [34]. Summing up, the Rich-Club is basically made up of areas involved in higher cognitive tasks devoted to planning and integration. The prominent role of the aforementioned regions in the cortex activity is unveiled from our network perspective in terms of a Rich-Club leading the path to synchronization. Our proposal, after this observation, is to investigate the evolution of synchronization in the cat cortex by tracking the transient of five modules corresponding to the anatomico-functional areas (S-M, F-L, Aud, Vis) and the Rich-Club as a separate (but interrelated) functional entities.

## Materials and Methods

### Cortico-cortical network of cats' brain

After an extensive collation of literature reporting anatomical tract-tracing experiments, Scannell and Young [8],[9] published a dataset containing the corticocortical and cortico-thalamical projections between regions of one brain hemisphere in cats. The connections were weighted according to the axonal density of the projections. Connections originally reported as *weak* or *sparse* were classified with 1 and, the connections originally reported as *strong* or *dense* with 3. The connections reported as *intermediate* strength, as well as those connections for which no strength information was available, were classified with 2, see Figure 1(b). Here we make use of a version of the network [10] consisting of 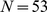 cortical areas interconnected by 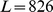 directed corticocortical projections.

### Rich-Club areas

A key factor of the hierarchical organization of the corticocortical network of the cat is that the hub areas (those with the largest number of projections) are very densely connected between them [16]. The Rich-Club phenomenon [30], [31] is characterised by the growth of the internal density of links between all nodes with degree larger than a given 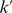, referred as 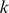-density, 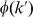:
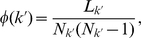
(5)where 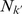 is the number of nodes with 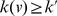 and 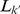 is the number of links between them. As 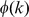 is an increasing function of 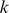, a conclusive interpretation requires the comparison with random surrogate networks with the same degree distribution. The question is then whether 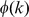 of the real network grows faster or slower with 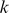 than the expected 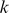-density of the surrogate networks. If 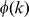 grows slower, it means that the hubs are more independent of each other than expected.

**Figure 8:**
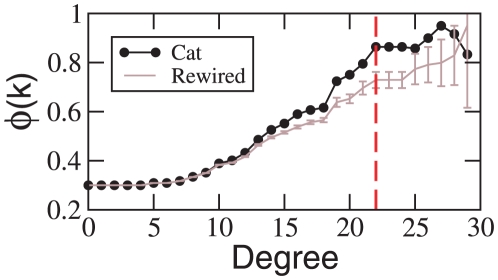
K-density of the corticocortical directed network of the cat 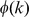, compared to the expectation out of the surrogate ensemble. The largest difference occurs at 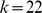 (vertically dashed line) giving rise to a Rich-Club composed of eleven cortical areas.

In our case, the network is directed but the input degree 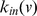 and the output degree 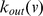 of the areas are highly correlated. Hence, we compute the 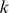-density of the corticocortical network of the cat, 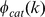, considering the degree of every area as: 
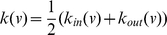
. The result is presented in Figure 8 together with the ensemble average 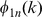 of 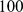 surrogate networks. At low degrees 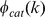 follows very close the expectation, but for degrees 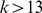, 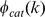 starts to grow faster. The largest difference occurs at 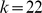, comprising of a set of eleven cortical hubs of the four modalities: visual areas *20a*, *7* and *AES*; auditory area *EPp*, somatosensory-motor areas *6m* and *5Al*; and fronto-limbic areas *Ia*, *Ig*, *CGp*, *CGa*, *35* and *36*.

### Numerical simulation details

We integrate the Kuramoto equations, see equation (1), using a fourth order Runge-Kutta method with time step 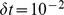. The system is set up by randomly assigning the initial conditions 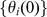 and the internal frequencies 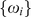 randomly in the intervals 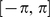 and 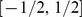 respectively. The integration of the Kuramoto is performed for a total time 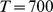. After a transient time of 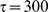 we start the computation of the different dynamical measures such as the order parameters 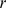 and 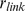.

### Synchronization order parameters

The dynamical coherence of the population of 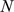 oscillators (areas) is measured by means of two different order parameters 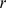 and 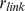. The first one is obtained from a complex number 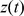 defined as follows:
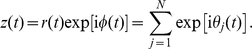
(6)The modulus of 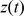, 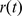, measures the phase coherence of the population while 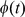 is the average phase of the population of oscillators. Averaging over time the value of 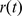 we obtain the order parameter 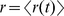.

The second order parameter, 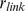, is measured looking at the local synchronization patterns, allowing for the exploration of how global synchronization is attained. We define 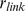 by measuring the degree of synchrony between two connected areas 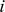 and 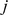:
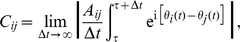
(7)where 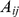 is the adjacency matrix of the network, being 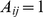 when 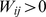 and 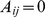 otherwise. Each of the values 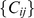 are bounded in the interval 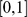, being 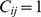 when the connected areas 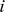 and 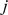 are fully synchronized and 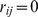 when these areas are dynamically uncorrelated. Note that for a correct computation of 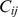 the averaging time 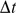 should be taken large enough (in our computations 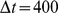) in order to obtain good measures of the degree of coherence between each pair of areas. Since 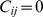 for the areas that are not physically connected we construct the 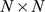 matrix 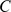 and define the global order parameter 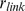 as follows:
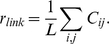
(8)Therefore, the parameter 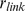 measures the fraction of all possible links that are synchronized in the network.

In the more general case in which all possible pairs of areas are taken into account to compute the average synchronization between cortical areas, Eq.(7) and Eq.(8) can be rewritten as:
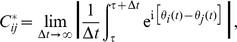
(9)and
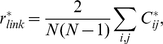
(10)respectively. Note that 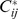 and 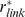 account for the degree of synchronization between areas 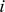 and 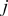 regardless of whether or not they are connected.

### Defining the average synchronization between areas

To label two areas 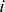 and 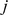 as synchronized or not one has to analyze the matrix 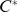 and construct a filtered matrix 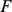 whose elements are either 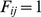 if 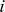 and 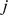 are considered as synchronized or 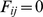 otherwise. From the computation of 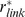, equation (10), one knows the fraction of all possible pairs of areas that are synchronized. Therefore, one would expect that 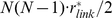 elements of the matrix 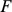 have 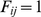, while the remaining elements are 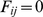. The former elements correspond to the 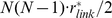 pairs with the largest values of 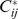.

In order to measure the average degree of synchronization between pairs of areas one have to average over different 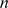 realizations using different initial conditions 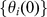 and different internal frequencies 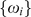 (typically we have used 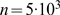 different realizations for each value of 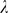 studied). To this purpose we average the set of filtered matrices 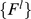 (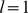,…,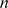) of the different realizations to obtain the average degree of synchronization between areas:
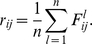
(11)In this way the value for 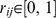 accounts for the probability that areas 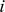 and 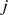 are considered as synchronized.
